# A review of rhegmatogenous retinal detachment: past, present and future

**DOI:** 10.1007/s10354-025-01085-9

**Published:** 2025-04-04

**Authors:** Jessica Xiong, Tuan Tran, Sebastian M. Waldstein, Adrian T. Fung

**Affiliations:** 1 Department of Ophthalmology, Westmead Hospital, Corner of Hawkesbury and Darcy Roads, Westmead, NSW Australia; 2 Save Sight Institute, The University of Sydney, Sydney, New South Wales Australia; 3 Sunshine Coast Health Institute, Griffith University, School of Medicine and Dentistry, Birtinya, QLD Australia; 4 Department of Ophthalmology, Sunshine Coast University Hospital, Birtinya, QLD Australia; 5 Department of Ophthalmology, Mistelbach-Gänserndorf Hospital, Mistelbach, Austria; 6 Westmead and Central Clinical Schools, Specialty of Clinical Ophthalmology and Eye Health, Faculty of Medicine and Health, The University of Sydney, Sydney, New South Wales Australia; 7 Department of Ophthalmology, Faculty of Medicine, Health and Human Sciences, Macquarie University, Macquarie, New South Wales Australia; 8 Karl Landsteiner University of Health Sciences, Krems, Austria

**Keywords:** History, Management, Vitrectomy, Pneumatic retinopexy, Pathogenesis

## Abstract

Retinal detachments are ophthalmic emergencies given their potential to become a permanently blinding disorder if left untreated. This review will outline the history, pathogenesis, clinical presentation, and current and future management of retinal detachments.

## History of retinal detachment

Over the last 100 years, retinal detachments (RD) have evolved from an untreatable, blinding disorder with unknown pathology to a condition with over 90% treatment success from a single surgery [1]. Jules Gonin played a pivotal role in the landscape of retinal detachment surgery by recognising that RDs were caused by retinal breaks. He first presented this to the Swiss Ophthalmological Society in 1919 [1–3]. Gonin went on to treat retinal breaks with ‘ignipuncture’, a form of thermal cautery performed with a hot pointed probe through a surgical sclerotomy. The patients then returned to specific positioning instructions for at least 1 week to allow the dependent subretinal fluid (SRF) to drain [1, 2, 4, 5]. Following these discoveries, various new methods were introduced over the years, of which three are still used today: pneumatic retinopexy, pars plana vitrectomy and scleral buckling.

## Definition

Retinal detachment is the separation of the retina off the retinal pigment epithelium (RPE) by SRF. The normal apposition of the retina to the RPE is crucial for visual function and comes about by means of a delicate interplay between active and passive forces of choroidal oncotic pressures and an RPE pump mechanism, thus creating a gradient between the two [6, 7]. The retina is responsible for converting light into an electrical signal through the process of phototransduction, which is then transmitted via the optic nerve to the visual cortex to form an image [7].

## Classification and pathogenesis

There are three major types of retinal detachment: rhegmatogenous, tractional and exudative. Although all types of retinal detachment are discussed, the scope of this review will be focused on rhegmatogenous retinal detachment (RRD), as this represents most retinal detachment cases.

### Rhegmatogenous retinal detachment

**Fig. 1:**
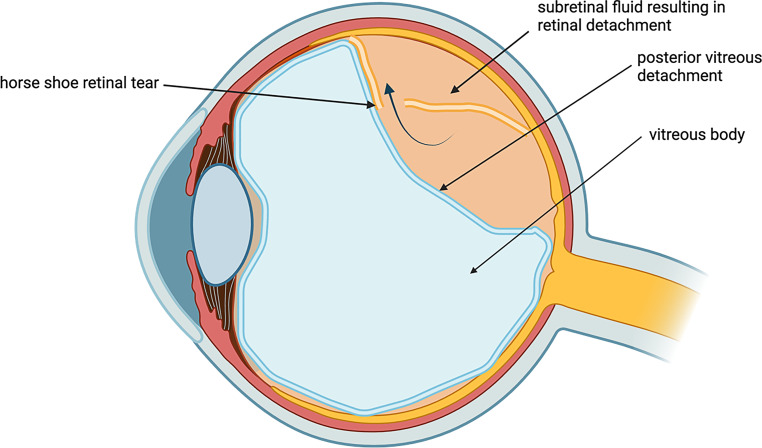
Posterior vitreous detachment generating traction, resulting in a retinal break and retinal detachment [11]

An RRD develops secondary to a full-thickness retinal break (hole or tear) which allows the accumulation of SRF [7]. Precursors of RRD include tractional forces on the retina, full-thickness retinal break and liquefied vitreous [8]. The retinal break is held open by tractional forces, allowing subretinal accumulation of liquefied vitreous [7, 9]. Without the presence of liquefied vitreous, an RRD will not form even with a full-thickness break ([10]; Fig. 1).

Most retinal detachments occur secondary to retinal breaks at the time of a posterior vitreous detachment (PVD) [6, 12]. Posterior vitreous detachment is a normal ageing process of the vitreous that occurs in everyone at some stage. The average age of onset of PVD is 60–70 years, with earlier onset associated with myopia or cataract surgery [13]. Ageing of the vitreous results in liquefaction of the gel (vitreous syneresis) [4]. A PVD often precedes RRD, as vitreous syneresis results in tractional changes on the retina producing a full-thickness retinal break where the posterior vitreous is adherent to the retina. As the PVD progresses from posterior to anterior, the vitreous may remain adherent to the flap of the break and a horseshoe tear results. If the vitreous traction is strong enough to result in avulsion of the retinal flap at the base, an operculated hole results, and the resultant tractional forces on the retina resolve [7]. Symptoms include sudden-onset floaters and flashes. The median time to retinal tear and RRD from initial PVD is 42 and 51 days, respectively, based on a recent large IRIS registry [14]. Thus, a dilated fundus examination with a repeat examination at 6 to 8 weeks is necessary to exclude retinal breaks after initial PVD.

More than 50% of acute symptomatic horseshoe tears may progress to RRD if not treated by prophylactic barrier laser retinopexy [15–17], which reduces the risk of RRD to less than 5% [18, 19]. Operculated tears make up 6–13% of retinal breaks and have a much lower risk of causing RRD. Studies of up to 80 patients with long-term follow-up have demonstrated none progressing to RRD [15, 20, 21], whereas one study demonstrated that one in six may progress to RRD [22]. Approximately 1.5% of asymptomatic atrophic holes without traction progress to subclinical or chronic retinal detachment [15, 23], accounting for 2.5–5% of all RRDs [24].

### Tractional retinal detachment

Tractional retinal detachment (TRD) occurs in the absence of retinal tears, where tractional forces pull on the surface of the retina via fibrovascular tractional bands, thus separating it from the RPE [4, 7]. Tractional bands attached to the retina either from the vitreous or within the retina itself contract, thereby lifting the retina from the RPE and leading to its detachment. TRDs can occur in a variety of proliferative retinal diseases but are most commonly associated with proliferative diabetic retinopathy [25]. Other pathologies include sickle cell retinopathy, proliferative vitreoretinopathy, retinopathy of prematurity and penetrating trauma [4, 25, 26].

### Exudative retinal detachment

Exudative retinal detachment (ERD), also known as serous retinal detachment, occurs from the accumulation of SRF without a retinal break or traction. These detachments are often associated with vascular, inflammatory or neoplastic conditions of the retina, RPE and choroid [27, 28]. With a normally functioning RPE, fluid is pumped out of the subretinal space and unable to collect. However, if fluid accumulation overwhelms the RPE pump, an ERD ensues [7, 29].

Causes of ERD include vascular diseases (central serous chorioretinopathy, hypertension, eclampsia, Coats disease, polypoidal choroidal vasculopathy) as well as inflammatory (Vogt–Koyanagi–Harada syndrome, sarcoidosis, tuberculosis), neoplastic (choroidal melanoma and metastases) and idiopathic causes (uveal effusion syndrome) [7, 30, 31].

## Demographics

An RRD can occur at any age, but the peak prevalence is 60–70 years, with a male predilection. Studies have supported a bimodal distribution, with the second most common age group peaking in 20–30-year-olds, particularly in highly myopic patients [32–34]. Observational studies across the United States, Europe and New Zealand estimated the incidence to be 12.17/100,000 people in a year, according to a recent systematic review and meta-analysis [32, 35, 36]. The incidences of RRD were highest in Europe (14.52/100,000), followed by the Western Pacific (10.55) and then the Americas (8.95). Ethnic variability was reported to be the highest in Caucasians (13.03/100,000), followed by Asians (9.88) and American Indians of Arizona (8.00). Importantly, a significant temporal trend was identified, with an annual RRD incidence increasing by 5.4/100,000 per decade from pooled data dating 1997–2019 [36]. This may be due to an aging population in Europe [37], closely followed by the Western Pacific, and an increased incidence of myopia and earlier cataract surgery [38–40].

## Risk factors for rhegmatogenous retinal detachment

An increased incidence of RRD is associated with many risk factors, both ocular (myopia, pseudophakia, lattice degeneration, aphakia, ocular trauma, uveitis, RRD in the fellow eye and retinal degenerative changes) and systemic (older age, male gender, positive family history and genetic disorders) [36, 37, 41, 42].

### Ocular risk factors

Myopia is a well-known risk factor for RRD, carrying a 10-fold increased incidence in people with over 3 dioptres of myopia and up to 50% of phakic RRD cases being myopic [4, 33, 35, 43]. Several factors explain the increased risk of RRD in myopic eyes, including the earlier onset of PVD (by 10 years); higher risks of retinal tears; lattice degeneration; and thinner, weaker retinas [7, 44, 45].

There is an increased risk of RRD in patients who have undergone cataract surgery, with up to 44% of those presenting with RRD being pseudophakic [46]. This risk is 0.5–0.6% after cataract surgery, 10 times higher than the general population [47–49]. In a recent IRIS Registry analysis of over 3,000,000 patients, post cataract surgery, RRDs occurred in approximately 1 in 500 cataract surgeries aged > 40 years, within 1 year of surgery [50]. The presence of lattice degeneration (weaker areas of the retina) resulted in the highest odds for RRD and retinal tear after cataract surgery [50, 51]. Younger age at cataract surgery was associated with a higher risk of RRD [52]. Complicated cataract surgery also increases the risk of retinal detachment. Specifically, studies have demonstrated that posterior capsule rupture during cataract surgery significantly increases the risk of RRD, with an increased hazard ratio of 12.83 compared to routine cataract surgeries [53, 54]. The pathogenesis of RRD following cataract surgery has been suggested to be due to the changes in vitreous anatomy following the replacement of a large cataractous lens, with a lower-volume intraocular lens. Additionally, transmitted tractional forces to the vitreous secondary to changes in anterior chamber depth and ultrasound energy intraoperatively can contribute [55]. Thus, the presence of posterior vitreous detachment in older patients prior to cataract surgery may act as a protective mechanism against subsequent RRD by limiting the forces transmitted to the retina during cataract surgery [52, 55].

Other ocular risk factors for RRD include trauma, uveitis (particularly herpes simplex, herpes zoster and cytomegalovirus retinitis), previous retinal detachment in the fellow eye and other degenerative conditions such as retinoschisis [35]. Blunt trauma is associated with retinal dialysis, whereby the retinal breaks are located anterior to the vitreous base and close to the ora serrata, thus distinguishing it from a retinal tear [56]. Asymptomatic retinal dialysis has a high risk of progressing to RRD, particularly following ocular trauma [35, 57]. Idiopathic macular holes can also cause RRD in myopic eyes; however, this is rarely seen in emmetropic or hypermetropic eyes [6].

### Systemic and genetic risk factors

The strongest systemic risk factor for RRD is age, with the incidence of RRD being maximal in the 60–69-years age group [58]. Studies have demonstrated males as having a high risk of RRD, with up to twice the risk of females [58–60]. Vascular risk factors have been studied with weak associations to RRD. Blood pressure and obesity have been shown to be associated with RRD, particularly in non-myopes; however, larger studies must be performed to better determine vascular risk factors and their role in RRD [61].

Evidence supporting a genetic component in RRD includes differing population incidences (ethnic variability) and a family history of RRD. There are many syndromic disorders that are associated with RRD, including Stickler, Marshall, Kniest, Marfan and Knobloch syndromes [62–65]. Most cases of RRD with genetic syndromes are secondary to inherent ocular risk factors such as myopia, premature cataract development at a young age and lattice degeneration [44, 66].

## Symptoms and signs

Typical symptoms of RRD overlap with those of PVD and include photopsia (flashes of light), myodesopsias (floaters), peripheral visual field defects and decreased visual acuity (VA). Recent studies have demonstrated that amongst patients presenting with flashes and floaters, the incidence of retinal tears or RRD is 14.3%. Reduced VA increases the likelihood of RRD fivefold in patients presenting with PVD [67].

Asymptomatic retinal detachments are often discovered incidentally during routine ophthalmic examinations; preoperative evaluations for refractive surgery; or in patients with mature cataracts, in whom visualisation of the posterior segment is limited. Asymptomatic retinal detachments were found in 0.039% of myopic eyes during preoperative assessment for laser refractive surgery in a study examining 6547 myopic individuals, thus emphasising the importance of thorough retinal evaluations in patients with high myopia [68]. Patients with mature cataracts should undergo B‑scan ultrasonography to exclude any retinal detachments, breaks and other posterior segment abnormalities that may be difficult to visualise through the lens opacity.

## Diagnosis

### Clinical examination

The diagnosis of RRD is clinical, requiring a dilated fundus examination facilitated by either slit lamp biomicroscopy or a binocular indirect ophthalmoscope. Anterior segment examination may be unremarkable, but it can reveal any underlying ocular comorbidities; risk factors for RRD (such as trauma and pseudophakia); or surgical considerations such as media opacification, inflammation, lens status, PVD status and poor dilation. Schafer’s sign is the detection of pigmented cells (RPE cells) in the anterior vitreous, which is strongly associated with a retinal break and should raise high suspicion for RRD [69].

**Fig. 2:**
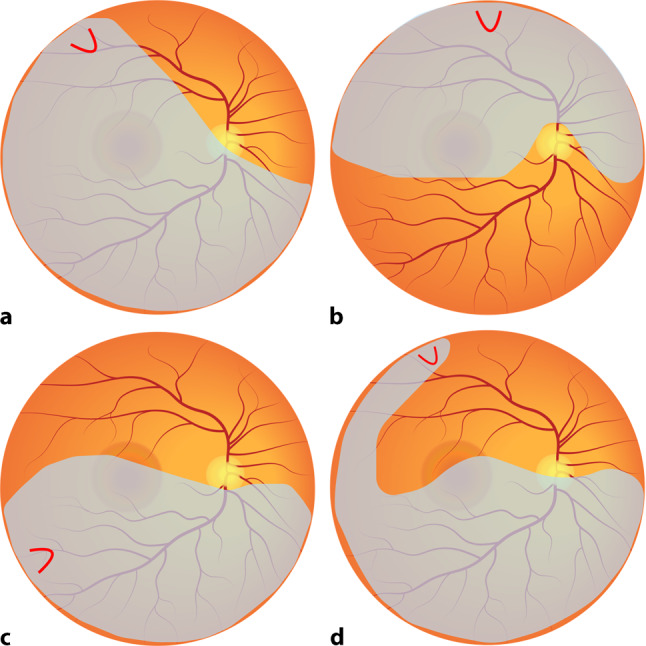
Summary of the Lincoff rules (blue = retinal detachment area and red = retinal break) **a** Superior temporal or nasal rhegmatogenous retinal detachment (RRD)—the primary break lies within 1.5 clock hours of the highest border; **b** total or superior RRD that crosses the 12 o’clock meridian—the primary break occurs at 12 o’clock or within 1.5 clock hours; **c** inferior RRD—the higher side of the detachment contains the break; **d** inferior bullous RRD—the primary break is usually a small hole close to 12 o’clock [11]

The key to examination in RRD is to search and identify all retinal breaks and establish the extent of the detachment. This can be performed by dilated fundus examination using indirect ophthalmoscopy and often requires external scleral depression or indentation to visualise the peripheral retina up to the ora serrata. This is performed with gentle pressure through the eyelids or directly on the sclera with topical anaesthesia. Key features to identify on examination include whether the macula is detached; the extent of retinal detachment in clock hours; the location, dimension and type of retinal tears; and presence or absence of a PVD, vitreous haemorrhage and proliferative vitreoretinopathy (PVR), all of which play a pivotal role in determination of the type of surgical intervention to undertake and its timing. The Lincoff rules have been well established for over 40 years to help in identifying the location of primary causative retinal breaks in RRDs. Figure 2 outlines a summary of the Lincoff rules [6, 70].

### Diagnostic imaging—colour photographs, OCT, B-scan ultrasonography

In most cases, the retinal detachment and retinal breaks can be identified on clinical examination without ancillary imaging. However, ultra-widefield (UWF) colour fundus photography can be helpful in documenting the extent of the RRD and in patients who may have difficulty tolerating an indented examination (such as in children).

Optical coherence tomography (OCT) is a noninvasive test that utilises light reflected to provide a cross-sectional image of the retinal layers [71]. It can assist in the detection of microstructural changes that are not always clinically evident, such as the presence of subretinal fluid at the macula in an RRD (“macula-on” or “macula-off”). These classifications have important implications for surgical timing and the visual prognosis. Macula-on RRDs typically have better VA on presentation and yield better visual prognoses. Surgery is typically performed within 24 h. Macula-off RRDs present with worse visual acuities and tend to have worse visual prognoses despite successful retinal reattachment [6].

Postoperatively, OCTs can also indicate important anatomic abnormalities after retinal reattachments, such as cystoid macula oedema, disruption of the ellipsoid zone/external limiting membrane, epiretinal membrane, disruption of retinal layers, retinal folds, outer retinal corrugations and residual subfoveal fluid [72]. These are important in explaining and predicting visual prognoses and metamorphopsia despite successful reattachment [71]. More recently, OCT angiography (OCT-A) has helped to predict visual prognosis after macula-off retinal detachment repairs, particularly due to the detection of an enlarged foveal avascular zone (FAZ) and reduced vessel density in the choriocapillaris [73].

Ultrasonography can also be utilised in the diagnosis of RRD, particularly when there is opaque media obstructing a clinical view of the fundus, such as with corneal opacification, dense cataract or vitreous haemorrhage [4, 74]. The retina is always attached to the optic nerve, and the B scan is performed in a dynamic fashion to distinguish between PVD, vitreous haemorrhage and RRD [4]. In late presentations and advanced stages of RRD, a funnel-shaped configuration may be seen due to a total PVR RD with only posterior attachment to the optic nerve. Retinal tears can also be visualised in the absence of retinal detachments [75].

## Differential diagnoses

Retinoschisis involves splitting of the retinal layers, which can be mistaken for retinal detachment. Retinoschisis appears translucent with prominent vasculature on widefield imaging, whereas retinal detachment appears opaque. Combined schisis–detachments exhibit mixed reflectivity patterns, and accurate diagnosis is essential for appropriate management [76]. Degenerative changes such as lattice degeneration and degenerative retinoschisis are common peripheral retinal conditions that can predispose to RRD. Lattice degeneration involves thinning and atrophy of the retina, often associated with retinal breaks, while degenerative retinoschisis can lead to schisis–detachment if outer layer breaks occur [18, 77].

Age-related macular degeneration (AMD), particularly in its exudative form, can mimic retinal detachment due to the presence of subretinal fluid and haemorrhage. Exudative AMD involves choroidal neovascularization, leading to fluid accumulation under the retina, which can be differentiated from RRD by the presence of drusen and pigment epithelial detachments [78].

Central serous chorioretinopathy (CSC) is another condition that can present with serous retinal detachment. Central serous chorioretinopathy is characterized by the accumulation of subretinal fluid due to choroidal hyperpermeability, typically affecting the macula and leading to visual disturbances. Differentiating CSC from RRD involves identifying the absence of retinal breaks and the presence of a characteristic leak on fluorescein angiography [78, 79].

## Natural course of retinal detachment

If left untreated, RRD most commonly progresses to near total or total detachment and blindness [80]. On occasion, the detachment can remain indefinitely and not progress with stable borders, in which case pigmented demarcation lines can form. This usually occurs in detachments that are asymptomatic and from small inferior breaks or dialyses [80, 81]. Rarely, the SRF from a superior break settles inferiorly and away from the break, resulting in the original break flattening [82]. Finally, and even more infrequently, spontaneous reattachment can occur due to the presence of a very small break and outstanding RPE pumping mechanism or closure of the break by scar tissue [4].

Other ocular sequalae from untreated retinal detachment include ocular hypotony and eventual phthisis. Longstanding RRD can also lead to neovascular glaucoma, resulting in a painful blind eye that requires evisceration. In less frequent instances, similar outcomes may arise following numerous failed surgical interventions in complex RRD scenarios [83]. Cataracts can also develop in untreated RRDs and occasionally proliferative vitreoretinopathy, although this is more common after surgical intervention with vitrectomy [84].

### Proliferative vitreoretinopathy

**Fig. 3:**
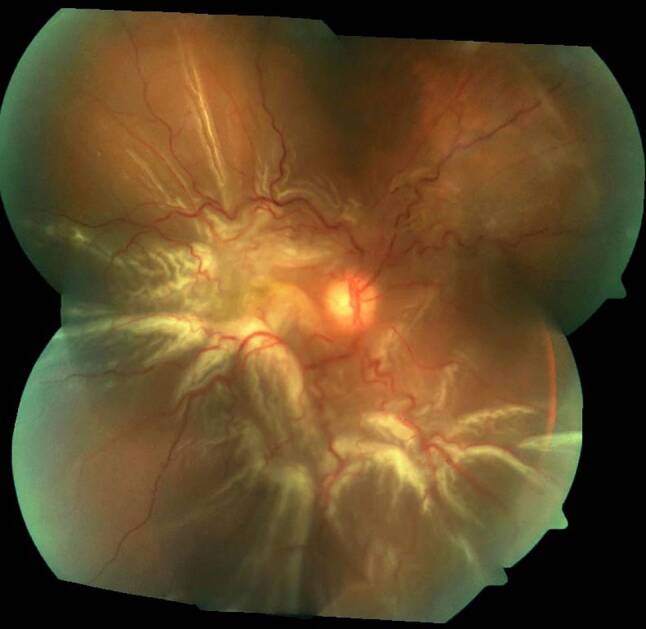
Colour fundus photograph of the right eye demonstrating proliferative vitreoretinopathy grade C retinal detachment with retinal star folds

The pathophysiology of proliferative vitreoretinopathy (PVR) has been extensively studied, with resultant formation of contractile proliferative cellular membranes on both the vitreous cavity and the retina [85]. Contraction of these membranes and intraretinal fibrosis can cause the retina to re-detach and fail to flatten. It is the most common cause of retinal detachment repair failure, with an incidence of 5–10% among all cases of RRD [86, 87]. Proliferative vitreoretinopathy grading was first recognised by The Retina Society Terminology Committee in 1983 and then updated in 1991 [86, 88, 89]. Grade A PVR was defined as the presence of vitreous haze and pigment clumps. Grade B PVR involves retinal wrinkling, rolled edges of the retinal break with retinal stiffness and vessel tortuosity. Grade C involves full-thickness retinal folds and/or subretinal bands and pathologic changes that can occur anteriorly, posteriorly or both. Grade C PVR was further differentiated into types 1–5, depending on the location of proliferation, type of contraction and extent in clock hours ([86, 89]; Fig. 3).

## Management

**Fig. 4:**
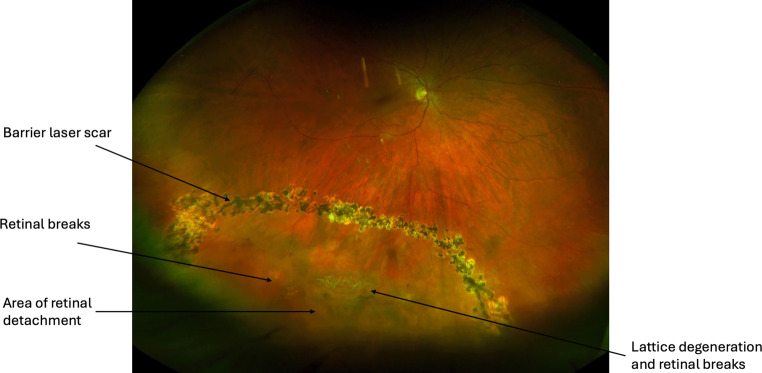
Inferior macula-sparing retinal detachment in a high myope with lattice degeneration and retinal breaks, barricaded by retinal laser

The aims of RRD management are to achieve retinal reattachment, seal all breaks and optimise visual outcomes. There are three main surgical interventions for RRD management: 1. pneumatic retinopexy (PnR), 2. scleral buckling (SB) and 3. pars plana vitrectomy (PPV). The choice of intervention is dependent on detachment location and extent, retinal tear location, presence of PVR, posturing compliance of the patient, lens status, need for postoperative air flight and surgeon preference [6]. For macula-sparing small localised acute RRD and chronic asymptomatic RRD, demarcation retinal laser photocoagulation alone can be effective as primary management ([90–92]; Fig. 4).

### Timing of surgery

Macula-on RRD is sight threatening and considered a vitreoretinal emergency requiring urgent intervention. It was also known that for macula-on RRD, repairs within 24 h of symptom onset resulted in significantly superior final vision compared to repairs after 24 h [93].

Acute macula-off RRD is also increasingly viewed as a vitreoretinal emergency, due to the potential for permanent visual loss and good postoperative visual recovery. Earlier studies suggested that there was no difference in final VA for macula-off RRD if operated on between days 1 and 7 [94]. However, more recent data have demonstrated that earlier intervention for macula-off RRD results in better final vision. A recent meta-analysis of surgical timing for macula-off RRD found that those who underwent repair on day 0–3 from symptom onset had superior final VA to those undergoing repair on days 4–7 [93]. In a prospective study of 2074 macula-off RRDs, the only risk factor that decreased the probability of achieving a postoperative VA of < 20/40 (0.5) was the duration of central vision loss. Repairing the RRD within the first 3 days was found to carry a better visual prognosis than later surgery [95]. Two separate studies found visual outcomes to be significantly better in patients with macula-off RRD undergoing treatment within 24 h of diagnosis compared to those treated between 24 and 72 h [96, 97]. Hence, studies support that more urgent intervention may improve visual outcomes in macula-off RRD.

### Pneumatic retinopexy

Treatment of RRD by PnR was first described by Hilton and Gizzard in 1986, as an outpatient procedure [98]. Following topical and/or subconjunctival anaesthesia, a small volume of expansile gas is injected into the vitreous space. The patient maintains a posture such that the intravitreal gas tamponades the retinal break whilst the RPE pump mechanism reabsorbs the SRF. The retinal breaks are sealed with cryotherapy before or laser retinopexy after the retina is reattached. PnR is suitable for detachments involving the superior retina with retinal breaks within 1 clock hour from 8 o’clock to 4 o’clock, with minimal media opacity, no PVR and in patients amenable to posturing [98].

**Fig. 5:**
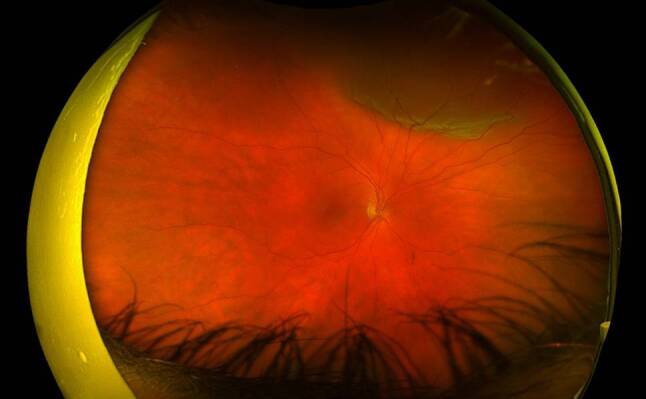
Right eye superonasal macula on retinal detachment

**Fig. 6:**
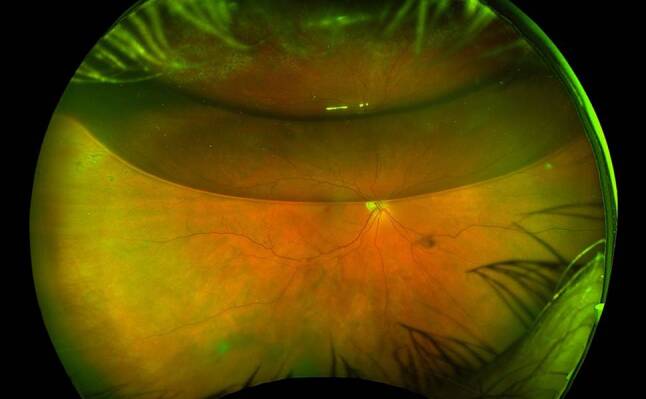
Flattened retina with superior laser over retinal break following pneumatic retinopexy (superior gas bubble visible)

Pneumatic retinopexy provided a simple outpatient alternative for management of RRD at a time where scleral buckling was the mainstay for RRD repair. It became increasingly attractive after a prospective multicentre randomised controlled trial had found superior VA in patients managed with PnR at 6 months and 2 years postsurgery when compared with scleral buckle, with no significant difference in primary success rates [99, 100]. More recently, one of the largest prospective randomised controlled trials, the Pneumatic Retinopexy versus Vitrectomy for the Management of Primary Rhegmatogenous Retinal Detachment Outcomes Randomized (PIVOT) trial, compared visual and anatomic outcomes in PnR versus PPV for management of primary RRD [101]. The anatomic success was greater in the PPV group at 12 months (93.2% vs. 80.8%); however, VA was superior in those undergoing PnR at every timepoint up to the endpoint of 12 months postintervention. The PnR group also demonstrated lower rates of vertical metamorphopsia and cataract at 12 months compared to the PPV group. Reduced vertical metamorphopsia is thought to be due to less retinal displacement after PnR compared with PPV [101]. This supports PnR as an effective initial management option for selected primary RRDs that fit the criteria, particularly in phakic patients (Figs. 5 and 6).

### Scleral buckle

Scleral buckling emerged in the 1950s and was considered the gold standard for surgical management for RRD for over 60 years [102]. It is most frequently performed in younger, myopic, phakic patients, who have localised RRD with small anterior holes or retinal dialyses and do not have a PVD or PVR. The surgery is most commonly performed under local anaesthesia (peribulbar, retrobulbar or subtenon block) but can also be performed under general anaesthesia. It involves localising the retinal break, cryotherapy, and either a local segmental or circumferential buckle to provide mechanical indentation and apposition of the neurosensory retina and RPE, thus relieving any traction [6]. This allows the retinal break to close and the SRF can be drained at the time of the procedure or left to spontaneously resorb over time.

**Fig. 7:**
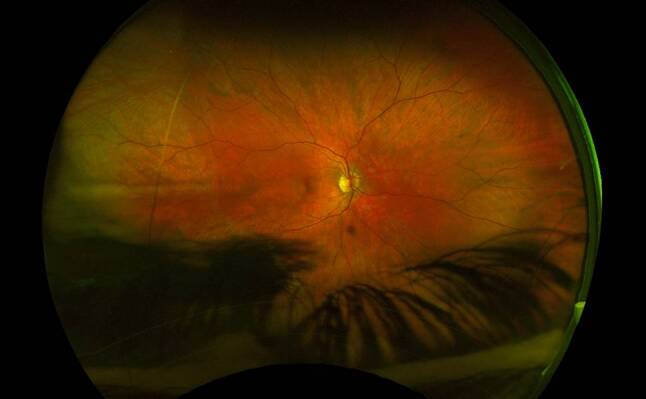
Inferior and temporal retinal detachment of the right eye, macula is on

**Fig. 8:**
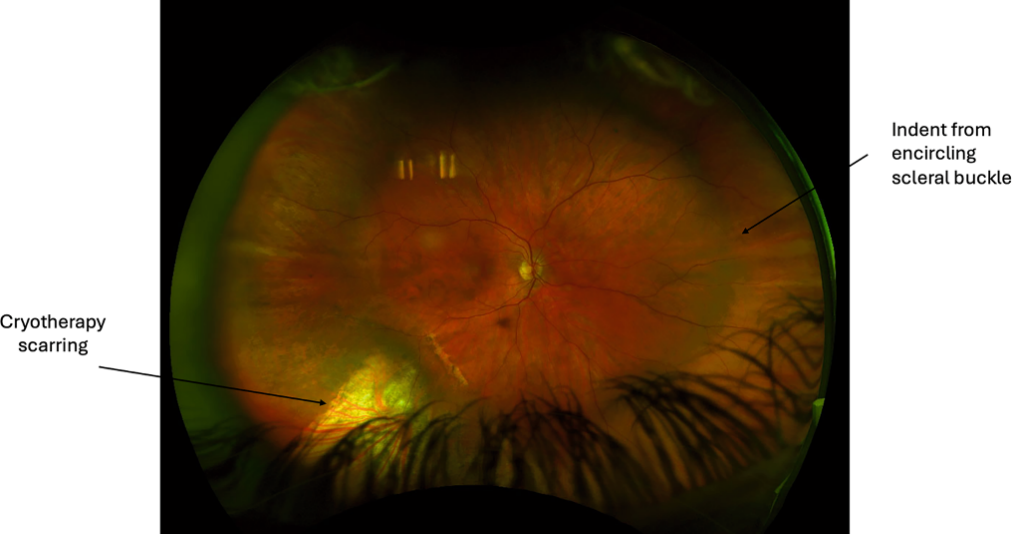
Reattached retina after scleral buckle surgery with cryotherapy

Scleral buckling has been found to be very successful as a primary repair technique for RRD, with success rates of up to 95% at 20-year follow-up [103]. However, with the advancement of minimally invasive small-gauge PPV and widefield intraoperative visualisation systems, scleral buckling has been used less in primary RRD repair. This may be due to SB being more time consuming and decreasing trainee exposure to the procedure [104]. The Scleral Buckling versus Primary Vitrectomy in Rhegmatogenous Retinal Detachment (SPR) study found that phakic patients undergoing SB had a greater VA improvement than after PPV, with less cataract progression. For pseudophakic patients, there was no VA difference between the two groups, but the primary anatomic reattachment rate was significantly higher in the PPV group [105]. In a recent meta-analysis comparing PPV with scleral buckling in 15,947 eyes, SB was found to have superior VA at the last follow-up; however, this was primarily due to phakic eyes developing cataracts after PPV [106]. Pars plana vitrectomy was found to carry a higher risk of cataract and iatrogenic retinal breaks. There was no difference in primary and final anatomic reattachment rates [106]. Overall, SB and PPV are considered comparable, with SB potentially more favourable in patients who are phakic ([105, 107]; Figs. 7 and 8).

### Pars plana vitrectomy

Pars plana vitrectomy has gained popularity since the 1970s, particularly with the advancement of ultra-widefield operating microscopes and micro-incisional (23- to 27-gauge) sutureless vitrectomy in the 2000s [108–110]. Indications for PPV include those in which SB is not appropriate, such as thin sclera, RRD with vitreous opacities limiting the retinal view, giant retinal tears, posterior breaks that cannot be reached with SB, presence of PVD, significant vitreoretinal traction that is unable to be relieved by SB and the presence of PVR [6]. It is often performed under a peribulbar block, retrobulbar block or general anaesthesia.

Vitrectomy utilises three transconjunctival sclerosotomies to allow entry of infusion fluid, light pipe, and vitrectomy cutter or other instruments into the eye. The vitrectomy guillotine cutter removes vitreous at up to 20,000 cuts per minute, internally relieving vitreoretinal traction on and around retinal tears. Proliferative vitreoretinopathy membranes can be peeled off the surface of the retina or removed from underneath the retina if present [111]. Once all traction is relieved, SRF is drained through the break(s) or a posterior retinotomy, and the retina is flattened. The retinal tears are then sealed with either laser retinopexy or cryotherapy, and the fluid in the eye is replaced with an intravitreal tamponade agent. Early positioning in the postoperative period is then adopted, depending on the location of the retinal detachment and type of tamponade. For macula-involving cases, face-down positioning for 24 h postoperatively is associated with a reduction in retinal displacement and subsequently binocular diplopia, particularly when adopted immediately after surgery [112]. In macula-on cases, positioning may be adopted to “support the break”, such as laterally (on either side) such that the tamponade opposes the primary break, increasing single-procedure reattachment rates [113].

Types of intravitreal tamponades for RRD include gas, oil and heavy liquids. Of the gases, sulphur hexafluoride (SF6) is often utilised, particularly for smaller superior breaks in RRDs that are unlikely to be complicated by PVR. Perfluoropropane (C3F8) is longer acting than SF6 and can be used when there are inferior retinal breaks for which postoperative positioning is more difficult. SF6 and C3F8 gases are naturally reabsorbed over time, with SF6 dissolving in 1–2 weeks and C3F8 over 6–8 weeks [114]. Silicone oil, however, does not spontaneously reabsorb and requires an additional surgery for its removal at 3–6 months to minimise the risk of retinal toxicity [115]. The silicone oil study found the use of silicone oil to be superior to SF6 but equivalent to C3F8 in PVR RRD in terms of greater reattachment rates and VA [116–118]. Thus, in PPV for PVR RRD, longer-acting gas such as C3F8 or silicone oil is often the tamponade of choice. For more challenging cases involving giant retinal tears complicated by PVR, heavy liquid (perfluorooctane, PFO), is commonly used as a safe, alternative temporary vitreous substitute to assist in vitrectomy surgery [119]. Being heavier than water, PFO is a useful agent to help flatten areas of stiffer retina affected by PVR. It can also be utilised intraoperatively in peeling tractional PVR membranes, thereby stabilising detached retina by exerting a downward force that flattens and holds the retina in place during surgery. This can facilitate safe and effective peeling of PVR membranes by providing a clear surgical field and preventing retinal slippage [120, 121]. However, PFO has a high retinal (photoreceptor) toxicity if it remains in the vitreous cavity for an extended period of time and needs to be removed after 1–2 weeks [115]. Thus, it is only used as a tamponade and left in the eye in exceptional cases.

**Fig. 9:**
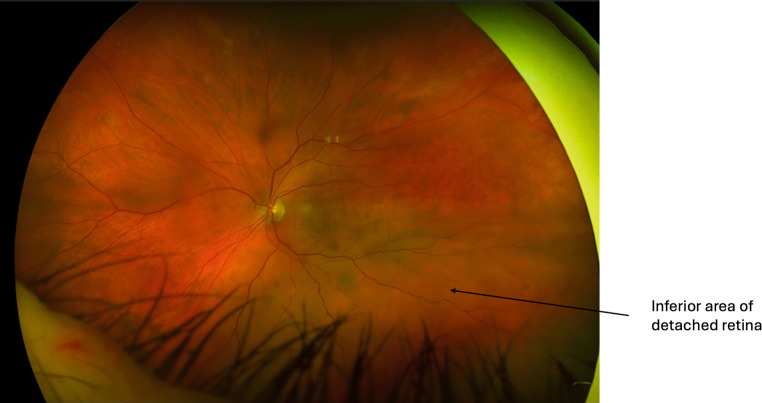
Left eye inferior retinal detachment involving the macula

**Fig. 10:**
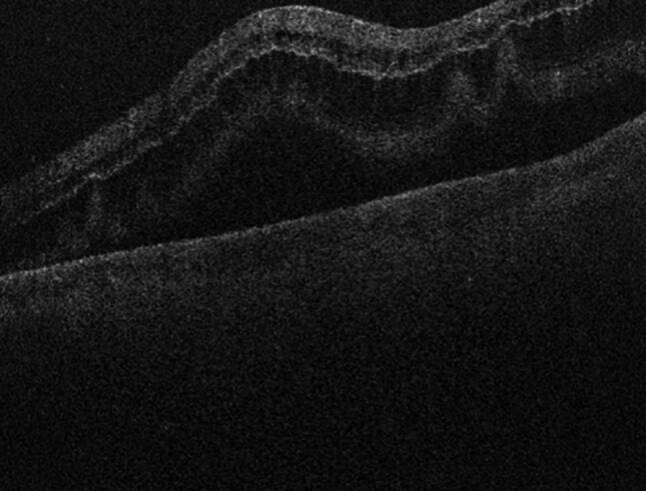
Optical coherence tomography image demonstrating macula involvement in the retinal detachment in Fig. 9

**Fig. 11:**
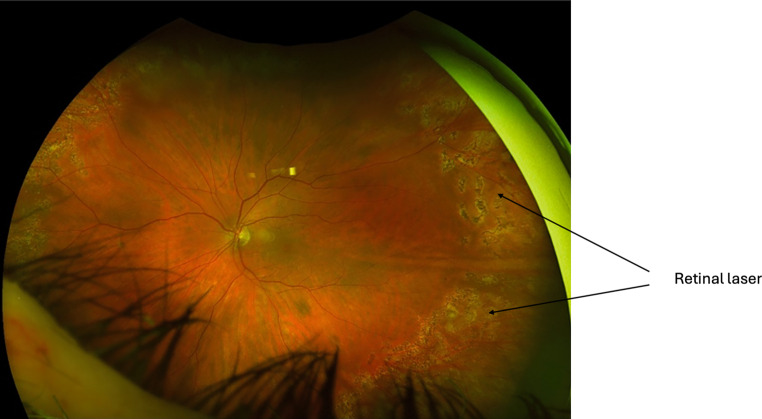
Post-vitrectomy surgery reattachment of the retina with 360-degree barrier laser

**Fig. 12:**
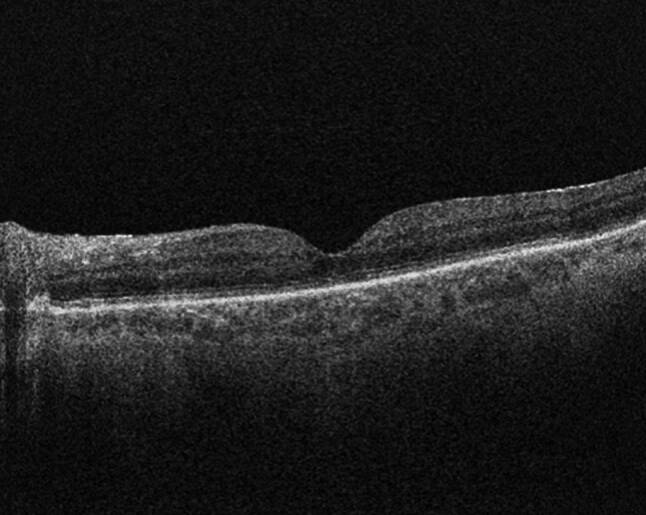
Optical coherence tomography image demonstrating reattachment of the macula after vitrectomy surgery

Pars plana vitrectomy has been very successful in treating RRD and is often the primary treatment of choice. Anatomic success rates have been reported of up to 96% for PPV, with low re-detachment rates of 6% reported [101, 105]. Cataract formation has been shown to be higher in phakic patients undergoing PPV compared to SB and PnR, affecting the final visual outcome. The PIVOT trial found VA to be improved by 1 and 17 Snellen lines following PPV for macula-on and macula-off RRD, respectively, at 1‑year follow-up [101]. The SPR study found better VA in phakic patients undergoing SB compared to PPV but found similar improvements in VA in pseudophakic patients undergoing SB and PPV [105]. Figs. 9, 10, 11 and 12 depict a 78-year-old male with left inferior macular-off RRD who underwent successful PPV.

### Combined pars plana vitrectomy and scleral buckling

**Fig. 13:**
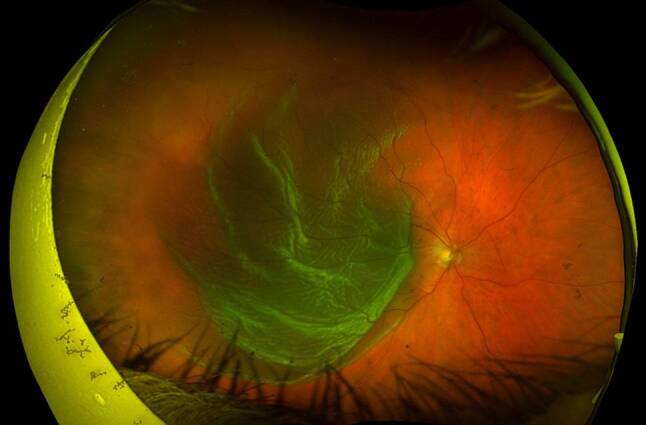
Right eye bullous temporal retinal detachment involving the macula

**Fig. 14:**
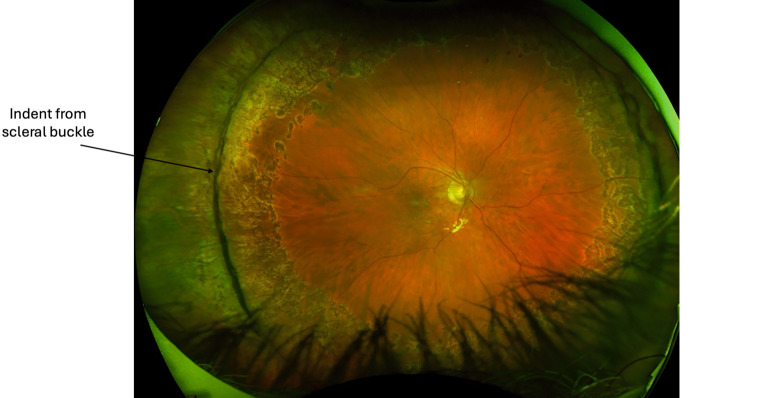
Right eye reattached retina after combined scleral buckle and vitrectomy with encircling buckle indentation, 360-degree barrier laser and silicone oil fill for travel

Pars plana vitrectomy and scleral buckling can be combined for more complex RRD cases or in cases with primarily inferior tear and detachment in which gas tamponade is less effective. A recent study from the Primary Retinal Detachment Outcomes (PRO) Study Group found greater single-surgery success with combined PPV/SB for inferior retinal breaks than with PPV alone (87.4% vs. 76.8%), particularly in phakic eyes (85.2% vs. 68.6%) [122]. This was similarly noted for both phakic and pseudophakic patients with moderately complex RRDs, where combined PPV/SB was found to have superior single-surgery anatomic success rates compared to PPV alone [46, 107]. Fig. 13 depicts a 60-year-old male with a right macular-off PVR RRD with Fig. 14 following PPV/SB/silicone oil tamponade.

### Postoperative care

With the use of gas tamponades, all patients should be informed that air travel and any activities that may result in a change in altitudes (such as air travel, travelling over mountains and scuba diving) are to be restricted until the gas has completely dissolved. This is due to the expansion of the volume of intraocular gas at lower atmospheric pressures, causing subsequent rapid elevation of intraocular pressure [123]. Similarly, whilst gas is still present, the use of nitrous oxide anaesthetics should also be avoided as nitrous oxide can diffuse more readily into air–fluid cavities, causing rapid expansion of the gas bubble and elevation of intraocular pressure that can lead to permanent ocular morbidity [124]. Silicone oil use requires later removal of oil as another procedure, which has associated risks of re-detachment, chronic retention leading to emulsification of oil, an increased rate of cataract formation and corneal decompensation [125, 126].

Postoperative head posturing is crucial in achieving optimal reattachment success and tamponading of retinal breaks. Retinal displacement has been shown to be reduced with early postoperative posturing, with retinal displacement being lower in patients who undergo immediate postoperative face-down positioning compared to positioning 10 mins after surgery [127]. This is particularly important for macula-off RRDs, which are at a higher risk of developing outer retinal folds and macula folds postoperatively, due to the structural changes in the detached retina and the presence of residual subretinal fluid after reattachment [128, 129]. Thus, the ability of a patient to follow appropriate posturing instructions should also be considered when deciding on the type of surgical intervention to optimise postoperative outcomes.

The development and progression of cataract following vitrectomy is a known adverse event, with most eyes requiring cataract extraction within 2 years of undergoing a PPV. The pathogenesis is thought to be due to the increased oxygen levels in the post-vitrectomy vitreous, which can no longer be neutralised by ascorbate that exists naturally in the vitreous after its removal [130]. Thus, the highly oxygenated environment results in oxidative damage to lens proteins and accelerates nuclear sclerotic changes [131, 132]. Furthermore, the use of intravitreal gas or oil tamponade results in posterior capsular opacities due to their direct contact with the lens capsule [133, 134]. Depending on the length of time and extent of contact with the gas, these opacities can spontaneously regress [135]. Hence, it is important to consider all risks and benefits, particularly in phakic patients, before undergoing a PPV for RRD repair.

### Complications

Complications vary between surgical procedures for RRD repair. For PnRs, complications involve traumatic cataract formation from capsular perforation, fish-egging of gas bubbles (formation of multiple smaller bubbles rather than one large gas bubble), subretinal gas, missed retinal tears, raised intraocular pressure, development of new retinal tears and vitreous haemorrhage [136–138]. In vitrectomy and scleral buckles, intraoperative complications include subretinal haemorrhage, hypotony and iatrogenic breaks [139, 140]. In the postoperative period, complications include endophthalmitis, re-detachment, elevated IOP, choroidal detachment, epithelial defect, cataract, cystoid macular oedema, macular pucker, PVR and epiretinal membrane formation [141–143]. Specific to scleral buckles, complications include buckle extrusion, diplopia and myopic shift [144].

## Prognosis

There are many factors that influence the visual and anatomic prognosis in RRD. Overall, there is a high anatomic success rate of up to 95%, with 70–90% achieving this in a single operation [35]. A final VA of 20/40 (0.5) or better is achieved in 90% of successfully repaired macula-on detachments. For macula-off detachments, only 50% achieve a VA of 20/50 (0.4), with worse outcomes if detachment has been present for at least 1 week [145, 146]. The most significant poor prognostic factors are macula detachment followed by the presence of vitreous haemorrhage, total retinal detachment, giant retinal tears, high myopia and the presence of PVR [146–148]. Additionally, other studies have found favourable anatomic and functional success in those with a preoperative VA of 20/50 (0.4) or better, RRDs that were less than total and retinal tears located anterior to the equator [149]. Postoperative causes of poor VA after successful anatomical repair include epiretinal membranes, cystoid macula oedema and foveal photoreceptor degradation in macula-off retinal detachments [145]. Although surgical interventions are often successful, in rare cases, multiple procedures may still result in total vision loss after retinal detachment repair.

### Risk of second eye retinal detachment

Patients who have experienced a rhegmatogenous retinal detachment (RRD) in one eye are at a significantly increased risk of up to 10% of developing RRD in the fellow eye, as pathologic vitreoretinal changes are frequently bilateral [18]. The presence of a posterior vitreous detachment (PVD) in the fellow eye at the time of initial RRD presentation is associated with a lower risk of subsequent RRD compared to eyes with an attached hyaloid. Prophylactic measures, such as laser retinopexy, may reduce the incidence of fellow-eye RRD, particularly in patients with high-risk retinal tears [150]. Close monitoring and patient education on the symptoms of RRD are crucial for early detection and management.

## Future of retinal detachment surgery

### Retinal glue

Endotamponading agents in PPV for RRD carry some disadvantages, including the requirement to position, secondary glaucoma, cataract formation and the need to undergo an additional surgery if silicone oil is chosen. Gas tamponade severely impairs patients’ vision postoperatively and should be discussed preoperatively with patients, particularly in the case of macula-on detachments or only eye detachments. Moreover, these tamponade agents have reduced efficacy in patients with inferior detachments. Novel use of retinal fibrin glue was first described in a pilot study with 5 patients, successfully sealing retinal breaks with no tamponade or postoperative positioning during RRD repair [151] A further study evaluated 26 patients undergoing RRD repair, finding fibrin glue to be a superior adhesive for sealing retinal breaks with no additional adverse effects, demonstrating it to be a considered alternative to gas or oil tamponade during PPV for RRD repair [152]. This demonstrates fibrin glue to be a potential safe and effective adjunct for surgical management of RRD. However, the evidence is still limited in this field, with limited case series, and further larger studies are required to confirm its safety and efficacy profile for widespread clinical use.

### Hydrogels

Vitreous substitutes currently employed in clinical practice have a range of complications such as oil emulsification, high intraocular pressure and cataract formation. An ideal vitreous substitute is transparent, biocompatible, viscoelastic and hydrophilic. Polymetric hydrogels exhibit these favourable characteristics, which make them suitable as an alternate vitreous substitute [153]. However, despite extensive research into various natural and synthetic polygels, none have been suitable for clinical use in RRD repair. Natural polymer-based hydrogels experience rapid degradation despite their biocompatibility and can thus only be used over a short time. Synthetic hydrogels, despite having better stability, have also been shown to be unsuitable [154–156]. More recently, there have been some breakthroughs in zwitterionic polymers which may show promise [154, 157, 158]. Further clinical studies into the favourable properties of vitreous hydrogels could reduce the postoperative complications associated with current tamponading agents.

### Neuroprotection

Patients with successful RRD repairs can still suffer poor visual outcomes due to photoreceptor apoptosis secondary to nutrient deprivation after prolonged separation from the RPE. Neuroprotective therapies are aimed to promote neuronal survival with the potential to protect against vision loss and retinal cell death [159]. Currently, a phase II clinical trial is underway for a small molecule, Fas inhibitor (ONL1204), in macula-off RRD [160]. The Fas receptor is a primary mediator of photoreceptor death and retinal inflammation in RRD, and targeting this receptor is thought to reduce photoreceptor apoptosis and improve the visual prognosis [161]. Ongoing research into neuroprotective mechanisms may therefore help to improve visual outcomes in patients with macula-off RRD.

### Management of PVR prevention

The most important risk factor for postoperative re-detachment is PVR. Despite this, there are still no proven therapeutic agents that can prevent or treat it. Preclinical studies exploring the use of corticosteroids, daunomycin and anti-vascular endothelial growth factor (anti-VEGF) have shown an inefficacy or significant ocular toxicity [162, 163]. Intravitreal methotrexate, a folate antagonist which exhibits antiproliferative properties has shown encouraging clinical results in some early clinical studies [164]. In 2022, Part 1 of the randomised, multicentre, phase III Gain Understanding Against Retinal Detachment (GUARD) trial reached its endpoint, reporting numerical superiority in re-detachment rates for intravitreal methotrexate over historical controls treated with slow-release dexamethasone [165]. Notably, there were no functional differences or safety concerns between the two groups [165]. More recently, a randomised control study of 43 patients found intravitreal methotrexate to be effective adjunct in managing PVR, with reattachment rates of 71% in the treatment group compared to 45% in the control group [166]. A recent systematic review also demonstrated higher reattachment rates and lower reoperation rates in methotrexate-treated eyes, with minimal adverse effects [167]. To improve PVR clinical outcomes, larger randomised clinical trials are required to better assess alternative therapies in combination with surgery for PVR-associated RRD.

### Heads-up 3D digital microscope

Since 2016, a three-dimensional heads-up display (3D HUD) visualisation system (NGENUITY; Alcon Inc., Fort Worth, Texas, USA) has provided an alternative approach to the traditional oculars microscope for intraocular surgery. Since then, multiple manufacturers have now offered this HUD system, such as the Artevo 800 (Carl Zeiss Meditec) and SeeLuma (Bausch and Lomb) systems. Surgeons wear polarised glasses and visualise the operative field on a high-definition screen that is projected from the operating microscope. Studies have demonstrated the 3D HUD to be safe and effective for vitreoretinal surgery in RRD with PVR, allowing for better surgeon ergonomics, lower endo-illumination requirements and better learning opportunities for staff and trainees [168, 169].

### Intraoperative OCT

Intraoperative OCT (IOCT) provides an intraoperative, noninvasive imaging of the retinal layers by utilising infrared light interferometry [170]. It can provide valuable feedback during vitreoretinal surgery and aid clinical decision making intraoperatively, particularly for RRDs that involve the macula. In a large prospective consecutive case series, IOCT images were successfully acquired in 98% of cases, with 29% of surgeons altering their surgical decisions during posterior segment surgery [171]. Intraoperative OCT is still a relatively new technique that presents exciting opportunities in vitreoretinal surgery.

## Conclusion

There has been significant improvement over the past 100 years in the diagnosis and management of RRD. It is considered an ophthalmic emergency given the potential to completely lose vision and, rarely, the eye. It is therefore critical, to accurately diagnose RRD, such that patients can undergo appropriate treatment in a timely fashion to maximise their visual potential. Vitrectomy surgery is the most common procedure for RRD, although both pneumatic retinopexy and scleral buckling maintain a role in certain circumstances. The choice of surgical technique is tailored to each case of retinal detachment, at the surgeon’s discretion. This variability underscores the ongoing search for an optimal surgical approach to RRD. The most common cause of re-detachment is PVR, for which new therapeutics are being studied. Future directions in RRD management aim to improve anatomic and visual outcomes, minimise complications, and improve surgeon ergonomics.
